# Inherited Tolerance in Cattle to the Apicomplexan Protozoan *Theileria parva* is Associated with Decreased Proliferation of Parasite-Infected Lymphocytes

**DOI:** 10.3389/fcimb.2021.751671

**Published:** 2021-11-05

**Authors:** Perle Latre de Late, Elizabeth A. J. Cook, David Wragg, E. Jane Poole, Gideon Ndambuki, Antoinette Aluoch Miyunga, Maurine C. Chepkwony, Stephen Mwaura, Nicholas Ndiwa, Giles Prettejohn, Tatjana Sitt, Richard Van Aardt, W. Ivan Morrison, James G. D. Prendergast, Philip Toye

**Affiliations:** ^1^International Livestock Research Institute (ILRI), Nairobi, Kenya; ^2^Centre for Tropical Livestock Genetics and Health, Nairobi, Kenya; ^3^The Roslin Institute, University of Edinburgh, Edinburgh, United Kingdom; ^4^Centre for Tropical Livestock Genetics and Health, Edinburgh, United Kingdom; ^5^Ol Pejeta Conservancy, Nanyuki, Kenya

**Keywords:** *Theileria parva*, tolerance, proliferation, parasitosis, pyrexia

## Abstract

*Theileria parva* is the causative agent of East Coast fever and Corridor disease, which are fatal, economically important diseases of cattle in eastern, central and southern Africa. Improved methods of control of the diseases are urgently required. The parasite transforms host lymphocytes, resulting in a rapid, clonal expansion of infected cells. Resistance to the disease has long been reported in cattle from *T. parva*-endemic areas. We reveal here that first- and second-generation descendants of a single *Bos indicus* bull survived severe challenge with *T. parva*, (overall survival rate 57.3% compared to 8.7% for unrelated animals) in a series of five field studies. Tolerant cattle displayed a delayed and less severe parasitosis and febrile response than unrelated animals. The *in vitro* proliferation of cells from surviving cattle was much reduced compared to those from animals that succumbed to infection. Additionally, some pro-inflammatory cytokines such as IL1β, IL6, TNFα or TGFβ which are usually strongly expressed in susceptible animals and are known to regulate cell growth or motility, remain low in tolerant animals. This correlates with the reduced proliferation and less severe clinical reactions observed in tolerant cattle. The results show for the first time that the inherited tolerance to *T. parva* is associated with decreased proliferation of infected lymphocytes. The results are discussed in terms of whether the reduced proliferation is the result of a perturbation of the transformation mechanism induced in infected cells or is due to an innate immune response present in the tolerant cattle.

## Introduction

*Theileria parva* is a tick-transmitted, apicomplexan parasite whose natural host is the African buffalo (*Syncerus caffer*), in which it causes few clinical signs. The parasite is restricted to eastern, central and southern Africa with its distribution closely aligned to that of its principal vector, the brown ear tick *Rhipicephalus appendiculatus*. The parasite also infects domestic cattle in which it causes an often fatal, lymphoproliferative disease called East Coast fever (ECF) (Irvin and Mwamachi, 1983). A clinically distinguishable form of disease called Corridor disease occurs in cattle when the parasite is transmitted directly from buffalo through ticks to cattle, as opposed to the cattle-tick-cattle transmission responsible for ECF. A hallmark of Corridor disease is a very low parasitaemia (Neitz et al., 1955).

In 2009, it was estimated that ECF causes losses of $300m annually (Spielman, 2009). The only currently available interventions to prevent ECF are intensive acaricide treatment to reduce tick infestation, and administration of a live sporozoite vaccine called ITM, the Infection and Treatment Method (Di Giulio et al., 2009). Both of these methods have major drawbacks. Acaricides must be applied regularly, with farmers in one region reporting that they dip or spray their animals as often as every four days (Allan et al., 2021). Acaricide treatment also carries risks of environmental pollution and the development of resistance in tick populations (Abbas et al., 2014). Although the ITM vaccine provides life-long immunity after a single inoculation, the production and delivery of the vaccine is complex and expensive (Patel et al., 2016), and the vaccine it is yet to be taken up in most endemic areas. Thus, there is an urgent need for new control methods to combat ECF.

Cattle are infected by introduction of the infective sporozoite stage of the parasite during tick feeding. The sporozoites enter host lymphocytes and develop to the multinucleate macroschizont form. The lymphocytes undergo a reversible transformation, replicating in synchrony with the parasite, which results in a rapidly expanding population of infected cells. Infected cells are initially detected in the draining parotid and prescapular lymph nodes before dissemination to more distal visceral organs such as the lungs, liver, spleen, kidneys and intestinal tract.

Tolerance to *T. parva* infection has been described in cattle from endemic areas. (Barnett, 1968) and (Irvin and Morrison, 1987) reported that *Bos indicus* cattle from endemic areas were less susceptible to disease caused by *T. parva* infection than both European *Bos taurus* cattle and *B. indicus* cattle from non-endemic areas. These observations were confirmed in experimental studies in which the clinical reactions in cattle from endemic and non-endemic areas were compared following challenge with a sporozoite stabilate (Ndungu et al., 2005). The inherited nature of this tolerance became apparent in studies where calves born to resistant dams and susceptible bulls were shown to have an intermediate susceptibility to ECF (Guilbride and Opwata, 1963). These results, together with the observation that progeny of disease-resistant cattle which have been reared in a tick-free environment are also tolerant and that serum antibodies appear to play no role in immunity to *T. parva* (Muhammed et al., 1975), suggests that the tolerance seen in calves from endemic areas is not due to maternal immunity nor is an inherent property of all *B. indicus* cattle, but is due to selection for disease resistance in endemic areas. The ability of cattle from *T. parva*-endemic regions to withstand an otherwise lethal infection with the parasite raises the intriguing question of how this tolerance is mediated. The availability of a clearly defined pedigree of resistant cattle would facilitate attempts to answer this question.

Through a series of field studies reported here, we have identified a sire line (bull 3167) of Boran (*B. indicus*) cattle that shows an inherited tolerance to natural field challenge with buffalo-derived *T. parva* infection. This was manifested as increased survival rates in the both first- and second-generation progeny, compared to unrelated animals of the same breed. We analyzed several parameters associated with the parasitic and pyrexic response in these animals. The results show that the surviving animals underwent a delayed and less intense parasitosis and febrile episode compared to the unrelated animals that succumbed to the field challenge. Associated studies involving *in vitro* cultures of infected lymphocytes showed a much reduced rate of expansion of infected lymphocytes from the animals that survived field infection compared to those that succumbed to severe disease. The results suggest that the ability of these cattle to overcome an otherwise lethal *T. parva* challenge is linked to a reduction in the proliferative rate of infected lymphocytes.

## Materials And Methods

### Animal Care and Use

The study was undertaken using protocols approved by ILRI’s Institutional Animal Care and Use Committee (Approvals 2013.03, 2014.32, 2015.29, 2017.02 and 2018.10). In instances where animals were showing severe signs of disease, particularly sustained pyrexia or hypothermia, anorexia and recumbency, a decision was made by the principal investigators in the field to treat the animal with buparvaquone or to submit the animal to euthanasia. The investigators were blind to the relatedness of the animals to bull 3167.

### Field Studies

Details of the logistics of the five field studies have been provided previously (Sitt et al., 2015; Cook et al., 2021), and the studies are described briefly here. The initial study (2013) was designed as a vaccine efficacy study. The animals for the four later studies were not vaccinated and were transported directly from the ILRI research station in Machakos, a region largely free of *T. parva*, to the Ol Pejeta Conservancy (Kenya), which is considered endemic for *T. parva*. The study site for the four later studies (00°00.971’N, 036°55.171’E; altitude 1797m) was 9km NE of the 2013 site, and both sites were populated by buffalo and other wildlife but not other cattle. Animals were screened in advance to exclude any which were positive by ELISA for antibodies against *T. parva* (Katende et al., 1998) and, in the 2017 and 2018 studies, by p104 PCR (Odongo et al., 2010). During the studies, the animals were held in pens overnight, and clinical observations and sampling were undertaken before the animals were released to graze during the day. The relatedness of the animals to bull 3167 was established by genotyping (Wragg et al., in preparation). Researchers handling the animals in the field were unaware of which animals were progeny or unrelated. A summary of the general design of the field studies is presented in **Supplementary Figure 1**.

### Cause of Death

The most likely cause of death for the study animals was buffalo-derived *T. parva* infection, based on clinical signs and post mortem observations (Sitt et al., 2015; Cook et al., 2021). Routine post mortem observations included frothy exudate in the trachea and pulmonary oedema. Seroconversion to anti-*T. parva* antibodies was detected in 46 of the 47 surviving animals and in 42 of the 74 animals that succumbed.

### Lymph Node Parasitosis

Needle biopsies were taken by aspiration from parotid and prescapular lymph nodes from the first day of swelling to the end of the study, death or if the animal returned three consecutive negative samples. The same lymph nodes were not necessarily sampled continuously to minimize damage. For the first two studies, biopsies were taken every 1 – 3 days. For the last three studies, biopsies were taken every day, alternating between left and right sides of the animal. Giemsa-stained smears prepared from the lymph node biopsies were examined by microscopy for the presence of schizont-infected lymphocytes. For the last three studies where intensive sampling was undertaken, an Infection Index was determined by counting the number of infected lymphocytes. The Infection Index is expressed as the number of infected cells per 100 lymphocytes. Infection Index values were calculated for both the parotid and prescapular lymph nodes. The Infection Index intensity was calculated as the average Infection Index value over the days when an Infection Index value was recorded. The average values were obtained from both the parotid and prescapular readings for each animal in the respective groups.

### Pyrexia

Rectal temperatures were obtained from the surviving animals on each morning of the study with a digital thermometer. On a small number of occasions a recording could not be taken due to the temperament of the animal. A reading greater than 39.4°C was considered elevated and indicative of pyrexia. The pyrexia intensity was calculated as the mean increase above 39.4°C over the days on which an elevated temperature was recorded.

### Establishment of Cell Lines *In Vitro*

*In vitro* cell lines: Venous blood was collected into Alsever’s solution and peripheral blood mononuclear cells (PBMC) were isolated by density gradient centrifugation as described (Goddeeris and Morrison, 1988). The PBMCs collected prior to field exposure were cryopreserved in liquid nitrogen. For infection, the cells were suspended in complete RPMI culture medium (RPMI 1640 supplemented with 10% heat-inactivated FBS, 2mM L-glutamine, 100 units/ml penicillin, 50ug/ml streptomycin, and 5 x 10^-2^M 2-mercaptoethanol). Cells were infected by incubation with freshly dissected salivary glands from *R. appendiculatus* ticks fed on animals infected with *T. parva* Muguga, stabilate 3087, as previously described (Goddeeris and Morrison, 1988; Morrison et al., 1996). The glands were suspended in complete RPMI medium and ground in a glass tissue grinder at room temperature. The sporozoite mix was centrifuged and the supernatant containing sporozoites was resuspended to an estimated 2,000 infected salivary gland acini per ml. Equal volumes of sporozoites and cells (2 x 10^7^ PBMCs) were mixed and incubated at 37°C for 90 min with periodic mixing. The cells were centrifuged, washed once, and resuspended in culture medium as described. Cells were placed in T25 flasks and maintained by adding fresh culture medium every 2 to 3 days.

In all cases, enough infected cells were generated for all of the proliferation experiments outlined below. After infection a part of cells were kept in culture for Giemsa staining and trypan blue counting. The rest of infected cells were stained with the CellTrace violet dye (**Supplementary Figure 2**).

### Estimation of Numbers of Live Cells

The number of live cells in the *in vitro* cultures was assessed by trypan blue exclusion. An aliquot of cells was removed from culture, stained with trypan blue and counted in a hemocytometer every two days for eight days during the first week and on three occasions during the second week. The numbers of both live and dead cells were recorded. As the cultures were maintained by adding fresh culture medium, a dilution factor was taken into account when counting the cells to determine the total number of cells that would have been present in each culture and time point. For example, if half the medium was replaced, a dilution factor of 2 was used. If this was repeated before the next count, the dilution factor was 4.

### Estimation of Numbers of Live Infected Cells

To detect live, infected cells in culture, the cells were removed from culture and washed twice in PBS before being transferred into 96-well microplates. The infrared dye LIVE/DEAD™ Fixable Near-IR Dead Cell Stain (ThermoFisher Scientific) was used to identify dead cells for removal. The cells were incubated in the dye for 20 min at room temperature. After a further two washes, cells were fixed in 4% paraformaldehyde in PBS for 10 min at room temperature. The cells were washed once with PBS and incubated for 30 min with permeabilization buffer (FACS buffer, 0.1% saponin and 20% heat-inactivated goat serum). The plate was centrifuged and the supernatant was removed. The cells were stained with monoclonal antibody (MAb) IL-S40.2 which recognizes PIM, the polymorphic immunodominant molecule expressed on the schizont surface. After two washes, the cells were incubated with goat anti-(mouse IgG2a) antibody conjugated to phycoerythrin for 30 mins at 4˚C. After three washes, the cells were fixed in 1% paraformaldehyde and analyzed by FACS. Fluorescence data were acquired using a BD FACSCanto II flow cytometer (Becton Dickinson, Belgium) and analyzed using Flow Jo software (FlowJo, LLC, Oregon, USA)). Fluorescence data from 10^5^ cells were acquired for each sample.

### Estimation of Cellular Proliferation Rate by Dye Dilution Assay

The rate of cell division was examined by dye dilution analysis with CellTrace™ Violet (CTV) (ThermoFisher Scientific). The analysis relies on the incorporation of a fluorescent dye into the cells. As the cells divide, those in each successive generation have a decreased amount of the dye, enabling the detection of distinct generations. Examination of fluorescent histograms at successive time points allows the rate of appearance of generations, and thus the rate of proliferation of cells, to be determined.

As CTV can cause cell death (Kamm et al., 2020), we undertook an initial experiment with concavalin A-stimulated PBMC to determine an optimal concentration of CTV. The cells were labelled incubated at 5µM, 10µM and 15 µM CTV, and examined at six days after stimulation (results not shown). Based on the appearance of generational peaks, a CTV of concentration of 10 µM was used for the remaining experiments.

After infection, PBMC were resuspended at 1×10^7^ cells in PBS containing 10 μM of CTV, with immediate vortexing to ensure rapid, homogeneous staining of cells. The cells were incubated at 37°C for 20 min, protected from light. Any unbound dye was quenched by adding 5 times the original staining volume of complete culture medium to the cells and incubating them for 5 min at 37°C. Cells were pelleted by centrifugation and resuspended in fresh pre-warmed complete culture medium. An aliquot of the cells was checked by flow cytometry to confirm adequate uptake of CTV. The cells were maintained in culture for eight days and analyzed using flow cytometry every two days. Due to loss of detectable fluorescence by day 8, a new batch of infected cells that had been maintained in culture without CTV since the beginning of infection was stained with CTV, in order to analyze proliferation during the second week of infection.

### White Blood Cell Isolation for qPCR

Blood obtained by venipuncture of the jugular vein was transferred to a labelled falcon tube and three volumes of red blood cell lysis buffer were added. Tubes were incubated for 5 min at room temperature. Once the blood changed from opaque to translucent aspect, tubes were centrifuged at 1,100rpm (300 x g) for 5 minutes. The pellet was washed twice in PBS and centrifuged at 1,100 rpm for 5 minutes. After discarding the supernatant, white blood cells (WBCs) were re-suspended in 1.4 ml of TRI Reagent™ (Sigma-Aldrich), aliquoted into two labelled Eppendorf tubes, and stored at -80˚C.

### RNA Extraction

RNA was extracted from the isolated WBCs using the RNeasy mini kit (Qiagen, Germany) according to the manufacturer’s instructions. RNA was quantified using Qubit BR RNA assay kit (Invitrogen), following which cDNA was generated using GoScript™ Reverse Transcriptase kit (Promega).

### Quantitative PCR

The cDNA was used to perform qPCR using Luna^®^ Universal qPCR Master Mix (New England Biolabs) and the primers listed in **Table 1**. The bovine GAPDH gene was amplified in each sample to monitor the concentration of template mRNA. Up to 100ng of cDNA were added to a final volume of 20 µl reaction mixture containing Hot Start Taq DNA polymerase and each of the oligonucleotide primers at a final concentration of 0.25 µM. The qPCR was performed in an ABI 7500 real time PCR system with the following conditions: Holding at 50°C for 2 minutes, 95°C for 10 minutes, 40 cycles of 95°C for 15 seconds and 60°C for 1 minute. This was followed by a melt curve from 60°C to 95°C. The specificity of amplification was confirmed by melting point analysis with SYBR Green™ and by determining the size of the PCR products by gel electrophoresis. The relative levels of mRNA of the cytokine genes were assessed by determining the respective Ct values normalized by comparison to the GADPH result. The fold change in each cytokine mRNA was calculated by using the ddCT method (Livak and Schmittgen, 2001).

**Table 1:** Primer sequences use for qRT-PCR.

Name	Forward	Reverse
TGFβ1	GCCATACTGGCCCTTTACAA	CACGTGCTGCTCCACTTTTA
TGFβ2	CTGGATGCAGCCTATTGCTT	CTGGATGCAGCCTATTGCTT
Il1β	CAAGGAGAGGAAAGAGACA	TGAGAAGTGCTGATGTACCA
IL6	CATTAAGCGCATGGTCGACA	CTCCAAACCCAGCAAAGACC
TNFα	CGTGGACTTCAACTCTCCCT	GCAGTTCAGCTCCGTTTTCA
GAPDH	GGTGATGCTGGTGCTGAGTA	TCATAAGTCCCTCCACGATG

### Statistical Analyses

Survival was assessed using Kaplan-Meier survival curves and comparisons were made using log-rank tests. The parasitological, temperature and time-to-event data from the field studies were analyzed in R version 4.0.3 using linear regression. The effect of relatedness (1^st^/2^nd^ generation or unrelated) was assessed after adjusting for trial, sex and age main effects. One set of analyses assessed the differences among first- and second-generation survivors and unrelated deaths, while a second set of analyses assessed the differences between first- and second-generation deaths and unrelated deaths. In both sets of analyses, for two parameters (maximum Infection index and Infection index intensity), a trial by relatedness interaction term was also required. Paired contrasts were conducted adjusting for multiple comparisons using Tukey. Model assumptions were checked using a series of residual plots. Statistical analyses of the data from cell culture were performed with the GraphPad Prism version 8 software. Student’s unpaired t-test (Welch’s test), a student paired t-test with a two-tailed distribution or a Kruskall-Wallis/Wilcoxon tests were performed to compare each group of animals at each time point.

## Results

### Tolerance to Infection With *T. parva* in a *Bos indicus* Pedigree

The initial indication of tolerance among the related Boran animals was detected in a vaccine study undertaken in 2013 (Sitt et al., 2015), in which animals were exposed to a natural field challenge in an area of buffalo-derived *T. parva* challenge. Four first-generation progeny of a Boran bull (3167) had been randomly selected for the study. Three of the progeny were the only unvaccinated animals to survive, while the fourth offspring was one of three vaccinated animals to survive. The results prompted two further field studies in 2014 and 2015, comprising unvaccinated first-generation progeny of the same sire alongside unrelated animals of the same breed, and two similar studies in 2017 and 2018 examining second-generation offspring (the progeny of male first-generation descendants of sire 3167). In all, the five studies comprised 28 first-generation, 47 second-generation and 46 unrelated animals. The survival data for each study are shown in **Table 2**. In summary, 67.9% of the first-generation and 51.1% of the second-generation progeny survived the field challenge, compared to 8.7% of the unrelated cattle. (In these analyses, ‘survived’ refers to animals which survived for the period of the field study without clinical or therapeutic intervention, and ‘succumbed’ includes those animals which died or were treated or euthanized for animal welfare reasons.). Surviving animals were free of or showed few microscopically detectable parasites and were clinically normal by the end of the respective studies, indicating that they had resolved the infection

**Table 2:** Summary of survival outcomes for progeny and unrelated animals over the course of five field studies.

Year	1^st^ generation	2^nd^ generation	Unrelated
2013	3 (3)		9 (0)
2014	10 (4)		14 (1)
2015	15 (12)		10 (0)
2017		24 (17)	7 (1)
2018		23 (7)	6 (2)
Total	28 (19)	47 (24)	46 (4)
Survival rate	67.9%	51.1%	8.7%

The freestanding numbers represent the number of animals in the indicated group and the numbers in parentheses are the corresponding number of survivors. Only unvaccinated animals are included in the data for 2013.

**Figure 1:**
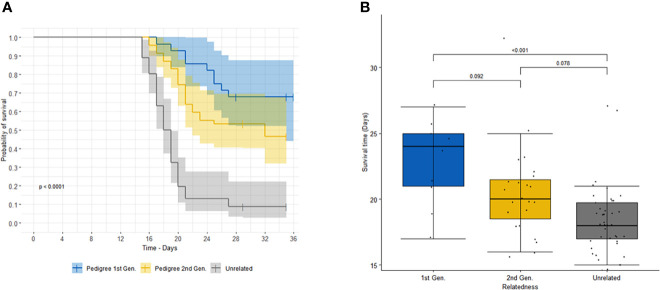
**(A)** shows the survival curves of first- (blue) and second- (orange) generation offspring of sire 3167 versus unrelated (grey) animals. Shaded areas correspond to 95% confidence intervals. **(B)** shows the comparison of survival time (or time to death) of animals that succumbed to infection for first- (blue) and second- (orange) generation offspring of sire 3167 and unrelated (grey) animals. The p-values for the specific paired comparisons from the linear regression (adjusted for multiple comparisons using Tukey) are shown above the boxplot.

This result is further illustrated in **Figure 1A**, which shows a significant difference in the survival curves between the combined progeny and unrelated controls (log rank test p=7 x 10^-13^). A similar difference in survival curves was observed when treating each generation of progeny as an independent group, along with the controls (log rank test p=3 x 10^-12^).

We also observed among the animals that succumbed that there was a longer time to death, euthanasia or treatment for progeny than for the unrelated animals (**Figure 1B**), with least square means (± s.e.) of 24.1 (± 1.11) days and 20.4 (± 0.86) days for the first-generation and second-generation progeny, respectively, and 18.7 (± 0.50) days for the unrelated group. (For animals which were treated or euthanized, the survival time was calculated up to the day of intervention.)

### *In Vivo* Field Observations

#### Surviving Progeny of 3167 Experienced a Delayed and Less Severe Lymph Node Parasitosis Than Susceptible Unrelated Animals

Macroschizont-infected lymphocytes were detected in the parotid and prescapular lymph nodes of all animals, except in one unrelated animal (2671) in the 2014 study. Nevertheless, this animal showed evidence of hyperplasia in the lymph nodes, developed a fever, died on day 16 and displayed macroschizont-infected cells in organ impression smears (Cook et al., 2021). To determine if there was any difference in the onset of parasitosis between the progeny survivors and the unrelated deaths, we calculated the least square means of the first day of detectable parasitosis (**Table 3**). It was clear that there was a significantly longer time for detection of parasitosis in both the first- (p ≤ 0.001) and second- generation (p = 0.006) survivors compared to the susceptible unrelated animals. There was no significant effect at the 5% level of age or sex of the animals on the time to parasitosis, and there was no significant interaction between the sire group and individual studies (p=0.08). For the last three studies, data were available to provide a quantitative estimate of the level of the parasitosis by determining the Infection Index (counting the percentage of infected cells in smears of lymph node aspirates), and the average intensity of the Infection Index values, the maximum Infection Index and the time to maximum Infection Index. Due to the low numbers of first-generation progeny for which data were available (2015 study only), the surviving progeny were analyzed as a single group. For both the Infection Index intensity and maximum Infection Intensity value, the least square means of progeny survivors were significantly lower (p ≤ 0.001 for both parameters) than those of the susceptible unrelated animals. Again, there was no effect of sex or age of the animals, although there was an interaction with the individual trials due to the relatively high Infection Index displayed by the small number (n=2) of unrelated animals in 2018 study. There was also a borderline significant difference between the progeny survivors and the susceptible unrelated animals (p = 0.055) in the least square means of the day on which the maximum Infection Index occurred. There was no significant effect of age or sex of the animals on the time to maximum Infection Index, and there was no significant interaction between the sire group and individual studies (p=0.78).

**Table 3:** Parasitosis and pyrexia data from the surviving offspring and the susceptible unrelated animals.

	First generation survivors (2013,2014,2015)		Second generation survivors (2017, 2018)		Unrelated deaths (all trials )		Overall Relatedness effect P value	Contrasts (P value)		
	lsm	s.e.	lsm	s.e.	lsm	s.e.		1^st^ vs. unrelated	2^nd^ vs. unrelated	1^st^ vs. 2nd
No. animals	19		24		42					
										
Time to pyrexia	15.2	(0.868)	15.5	(0.939)	12.9	(0.519)	0.003	0.037	0.053	0.974
Pyrexia intensity	0.81	(0.090)	0.73	(0.100)	1.19	(0.054)	<0.001	<0.001	<0.001	0.853
Max. temp.	40.8	(0.155)	40.6	(0.168)	41.5	(0.093)	<0.001	<0.001	<0.001	0.922
Time to max. temp. ^1^	16.9	(0.441)	17.0	(0.478)	15.7	(0.264)	0.003	0.034	0.060	0.986
Time to parasitosis^&,1^	13.0	(0.327)	12.9	(0.354)	11.6	(0.196)	<0.001	<0.001	0.006	0.985
	lsm		s.e.		lsm	s.e.	P value	1^st^ & 2^nd^ vs. unrelated		
No. animals	12		24							
Infection Index intensity^1,+^	0.31		(0.078)		1.07	(0.099)	<0.001	<0.001*		
Max. Infection Index^1,+^	1.01		(0.348)		4.00	(0.440)	<0.001	<0.001*		
Time to max. Infection Index^1,+^	18.1		(0.333)		17.0	(0.421)	0.055	0.055 (borderline)		

The values represent the least square means (lsm) and standard errors (s.e.) for each of the groups and are shown as days, Infection Index or °C. The Relatedness effect indicates the level of significance of the differences when all three groups were compared together (main effect) and was fitted after adjusting for trial1, age and sex (super-scripts indicate where the main effect was significant). The pairwise contrasts display the level of significance of the specific paired differences (adjusted for multiple comparisons using Tukey). ^&^Not including animal 2671 who died from T. parva infection but showed no ante mortem parasitosis. ^+^Analyses combine 1st and 2nd generation because of small sample size. *There was a significant (p < 0.001) trial x relatedness interaction due to a small number (n = 2) of unrelated animals with a high Infection Index in study 5.

The results show that both first- and second-generation surviving progeny of 3167 displayed a delay in the onset of and a less intense lymph node parasitosis than susceptible unrelated animals.

#### Surviving Progeny of 3167 Experienced a Delayed and Less Severe Pyrexia Than Susceptible Unrelated Animals

With one exception, all animals developed pyrexia (rectal temperature >39.4°C), with the earliest appearance being at day14 following exposure. The exception was a surviving, second-generation progeny in the 2017 study (animal 4150), whose maximum recorded temperature was 39.2°C. When four indicators of the pyrexic response were examined (**Table 3**), it was clear that pyrexia was slower to develop and less intense in the surviving progeny of 3167. Specifically, the least square mean time to the appearance of pyrexia was longer for both first- and second-generation animals (p=0.037 and 0.053, respectively) compared to susceptible unrelated animals. In addition, the mean time to maximum temperature was significantly longer for the first-generation animals (p=0.034) compared to the susceptible unrelated animals. For the second-generation animals, this interval was slightly longer again, but was marginally non-significant at the 5% level (p=0.06) due to a large standard error. We also observed that the mean maximum temperature and pyrexia intensity were significantly lower for the both first- and second-generation offspring (p ≤ 0.001), compared to the susceptible unrelated animals.

The results reflect those observed with the parasitosis observations, in that there was a delay in the onset of and a less intense pyrexia in both first- and second-generation surviving progeny of 3167 than susceptible unrelated animals.

#### Susceptible Progeny of 3167 Show a Level Of Disease Intermediate Between Surviving Progeny and Susceptible Unrelated Animals

We compared various survival, parasitological and pyrexic measurements from the susceptible progeny of 3167 with those from the unrelated susceptible animals (**Table 4**).There were several parameters that were significantly different between the susceptible first-generation progeny and unrelated animals, whereas other parameters, especially those related with time to detection of onset, were only marginally significant (0.05≤p≤ 0.10) or not significant. In contrast, the differences between second- generation and unrelated susceptible animals across all parameters were all only marginally significant (0.05≤p≤ 0.10) or not significant. The parameters related to Infection Index, which required combining the results from first- and second-generation animals, were highly significantly different between the susceptible progeny and unrelated animals, apart from that associated with time to maximum Infection Index. There were also significant differences between the susceptible first-generation progeny and the unrelated animals in the time between death and initial detection of parasitosis and pyrexia (p=0.002 and p ≤ 0.001, respectively), and death and maximum temperature (p=0.002). These intervals were not significantly different between second-generation animals and the susceptible unrelated animals. We also observed a significant difference in the time from maximum Infection Index to death (p=0.003) between the combined progeny and unrelated controls. The results suggest that those first-generation offspring that eventually succumbed to the field challenge nevertheless demonstrated some capacity to resist the clinical effects of *T. parva* infection.

**Table 4:** Survival, parasitosis and pyrexic data from the susceptible progeny and the susceptible unrelated animals.

	First generation deaths (Trial 2 – 3)		Second generation deaths (Trial 4 – 5)		Unrelated deaths (Trial 1 – 5)		Overall Relatedness effect P value	Contrasts (P value)		
	lsm	s.e.	lsm	s.e.	lsm	s.e.		1^st^ vs. unrelated	2^nd^ vs. unrelated	1^st^ vs. 2nd
No. animals	9		23		42					
Time to pyrexia	14.2	(0.931)	14.1	(0.721)	13.1	(0.421)	0.308	Overall effect non-significant		
Time from pyrexia to final day	10.0	(1.216)	6.7	(0.941)	5.4	(0.550)	<0.001	<0.001	0.496	0.144
Pyrexia intensity	0.79	(0.113)	1.03	(0.088)	1.16	(0.051)	0.004	0.005	0.468	0.288
Max. temp.	40.9	(0.193)	41.1	(0.149)	41.5	(0.087)	0.001	0.009	0.078	0.767
Time to max. temp. ^1^	17.1	(0.593)	16.3	(0.459)	15.8	(0.268)	0.078	Overall effect borderline sig.		
Time from max. temp. to final day	7.0	(1.041)	4.5	(0.806)	2.7	(0.470)	<0.001	0.002	0.155	0.221
Time to parasitosis^1,&^	12.8	(0.553)	12.7	(0.428)	11.6	(0.249)	0.009	0.075	0.076	0.992
Time from parasitosis to final day^1,&^	11.3	(1.19)	8.1	(0.924)	7.0	(0.538)	0.001	0.002	0.570	0.147
	lsm		s.e.		lsm	s.e.	P value	1^st^ & 2^nd^ vs. unrelated		
No. animals	3		23							
Infection Index intensity^1,+^	0.40		(0.111)		1.06	(0.120)	<0.001	<0.001		
Max. Infection Index^1,+^	0.74		(0.503)		3.87	(0.546)	<0.001	<0.001		
Time to max. Infection Index^1,+^	17.1		(0.406)		16.9	(0.441)	0.786	Overall effect non-significant		
Time from max. Infection Index to terminal day^1,+^	4.0		(0.615)		1.2	(0.667)	0.003	0.003		

The values represent the least square means and standard errors (s.e.) for each of the groups and are shown as days, Infection Index or °C. Some of the data for the unrelated susceptible animals from **Table 2** are included for clarity. The Relatedness effect indicates the level of significance of the differences when all three groups were compared together (main effect) and was fitted after adjusting for trial1, age and sex (super-scripts indicate where the main effect was significant). The pairwise contrasts display the level of significance of the difference of the specific paired differences (adjusted for multiple comparisons using Tukey). ^&^Not including animal 2671 who died from T. parva infection but showed no ante mortem parasitosis. ^+^Analyses combine 1st and 2nd generation because of small number (n = 2) of unrelated animals with a high Infection Index in study 5.

In summary, the analysis of clinical data from the field studies demonstrates that the tolerance exhibited by the progeny of 3167 is associated with significantly fewer and a delayed appearance of infected lymphocytes in peripheral lymph nodes and a delayed and less severe pyrexia.

### *In Vitro* Results

#### Cultures of Lymphocytes From Susceptible Cattle Show a More Rapid Increase in Viable Cells Compared to Those From Surviving Animals

**Figure 2:**
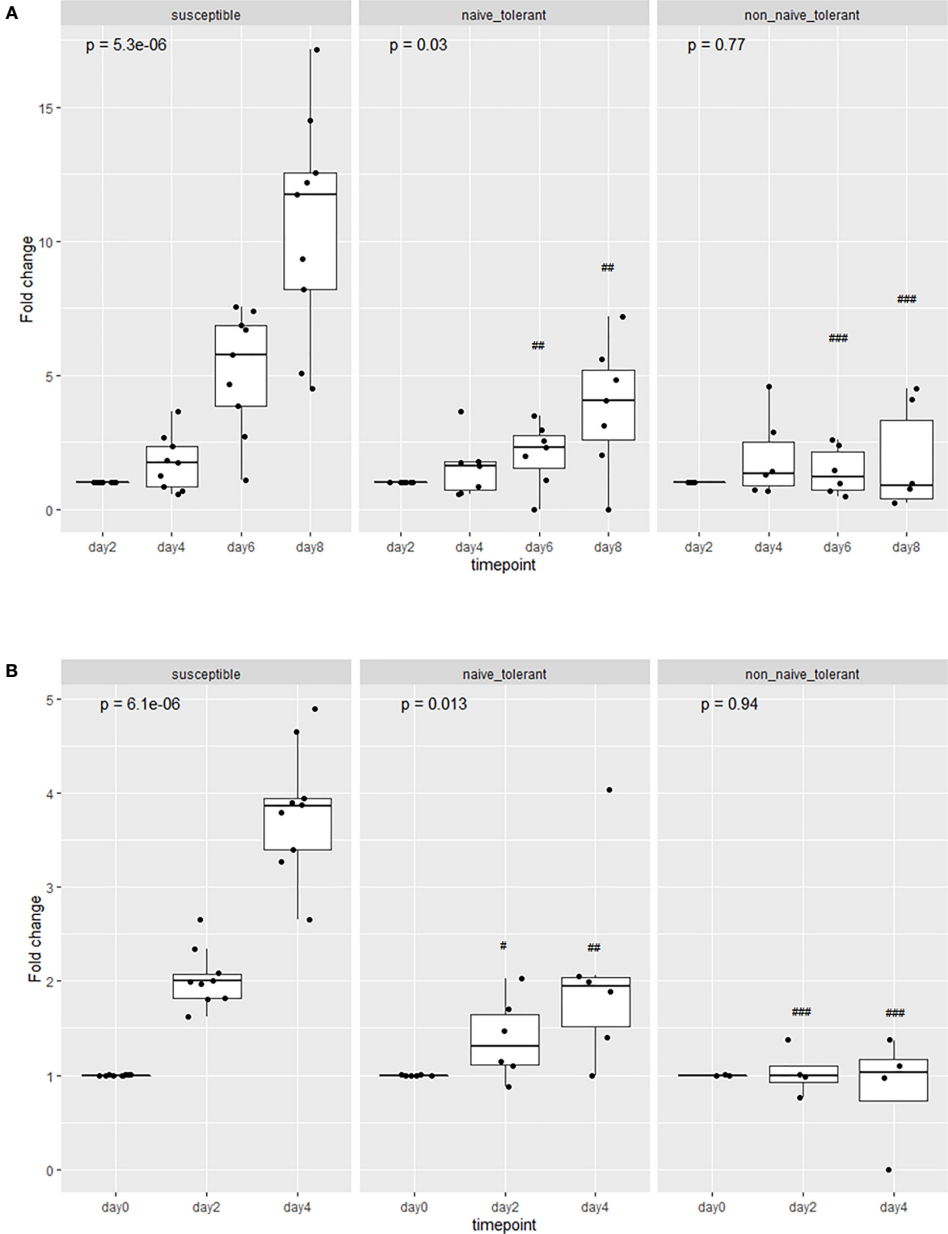
Proliferation of cells from susceptible and tolerant animals infected *in vitro*. The cells were collected from tolerant animals both before (naive) or after (non-naïve) the field study. The proliferation is represented as the group means of the fold changes in the number of live cells in each culture. Analyses were performed during the first **(A)** and the second week **(B)** of culture following infection. In panel **(B)**, day ‘0’ refers to the first day of counting (8 days after infection). The p values represent the probability of a difference in the fold change at different days within each group (Kruskal-Wallis test). The probability of a difference in fold change at the same day between susceptible and tolerant populations is shown as ^##^p < 0.01; ^###^p < 0.001 (Wilcoxon test).

PBMCs collected and cryopreserved before field exposure from seven surviving and nine susceptible animals (**Supplementary Table 1**) from the 2018 field study were infected *in vitro* with *T. parva* sporozoites to compare the kinetics of infection. The number of live cells was assessed by trypan blue exclusion, every two days for two weeks following infection. The results revealed only a modest increase in the number of live cells in cultures from the tolerant animals, compared to a statistically greater increase (p ≤ 0.01) in cultures from the susceptible animals during the first (**Figure 2A**) and second (**Figure 2B**) weeks of culture. The difference was statistically significant from day 6 of culture.

#### Cultures of Cells From Surviving Cattle Contain Fewer Viable Infected Lymphocytes Compared to Those From Susceptible Cattle

**Figure 3:**
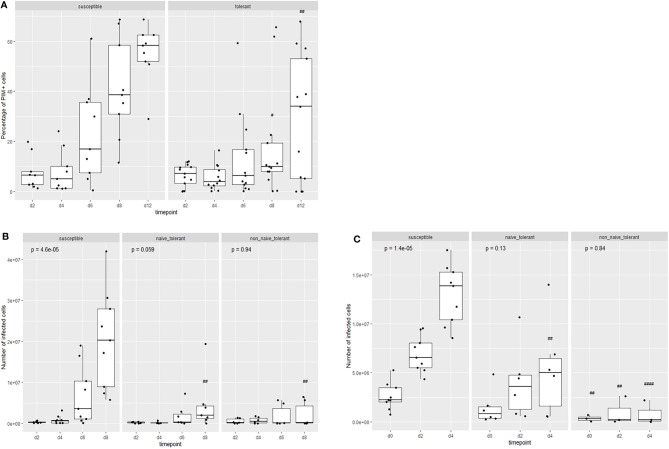
Proliferation of live infected (PIM+) cells from tolerant and susceptible animals. Change in the proportion **(A)** and numbers **(B, C)** of live infected cells in cultures from tolerant and susceptible animals during the first **(B)** and second **(C)** week of culture after infection *in vitro*. The results are the means of cell lines from 13 surviving animals and 9 susceptible animals. The p values represent the probability of a difference in the values at different days within each group (Kruskal-Wallis test). The probability of a difference in values on the same day between susceptible and tolerance populations is shown as ^##^p < 0.01; ^####^p < 0.0001 (Wilcoxon test).

The trypan blue method used above does not distinguish between infected and non-infected cells. To compare the rates of proliferation between infected cells from surviving and susceptible animals, infected cells were identified with a fluorophore-labelled MAb directed against the schizont surface antigen, PIM (Polymorphic Immunodominant Molecule), and counted by flow cytometry. **Figure 3A** shows that the proportion of PIM^+^ cells increases over time but remains lower in cultures from tolerant animals. The increase in the proportion of PIM^+^ cells in cultures from susceptible cattle coincides with the increase in live cells as measured by trypan blue. When the absolute numbers of live, infected cells were compared (**Figures 3B, C**), it was clear that there were significantly fewer live, infected cells in the cultures from surviving cattle compared to those from susceptible ones. The ‘outlier’ surviving animal (4807) was one of the four unrelated animals to survive the field exposure.

#### Increased Rate of Cell Death in Cultures From Surviving Animals

The previous results suggest that there was a comparatively smaller increase in the numbers of live infected cells in the cultures derived from tolerant animals. This may be due to a suppression in the proliferation of these cells, or an increase in the rate of cell death, or both. If the difference is due only to a lower rate of proliferation with no increase in the rate of cell death, the percentage of dead cells should be similar in the cell cultures from surviving and susceptible animals. To assess this, Giemsa-stained cells from the cultures were examined to assess morphological features, especially indicators of host cell death, during culture. We initially examined a negative control culture of uninfected PBMCs. This showed that the number of dead cells decreased considerably in two days (from about 7.8 million to 1.03 million cells) and disappeared completely in four days (**Supplementary Figures 3A** and **4**). As displayed in **Supplementary Figure 3B**, the examination of cells from a susceptible animal (as represented by animal 4819) revealed a steady accumulation of apparently healthy cells with easily visible, intact macroschizonts. In contrast, cells from a surviving animal (4199) showed signs of host cell death and disintegration with fewer healthy infected cells as the culture progressed (**Supplementary Figure 3C**).

**Figure 4:**
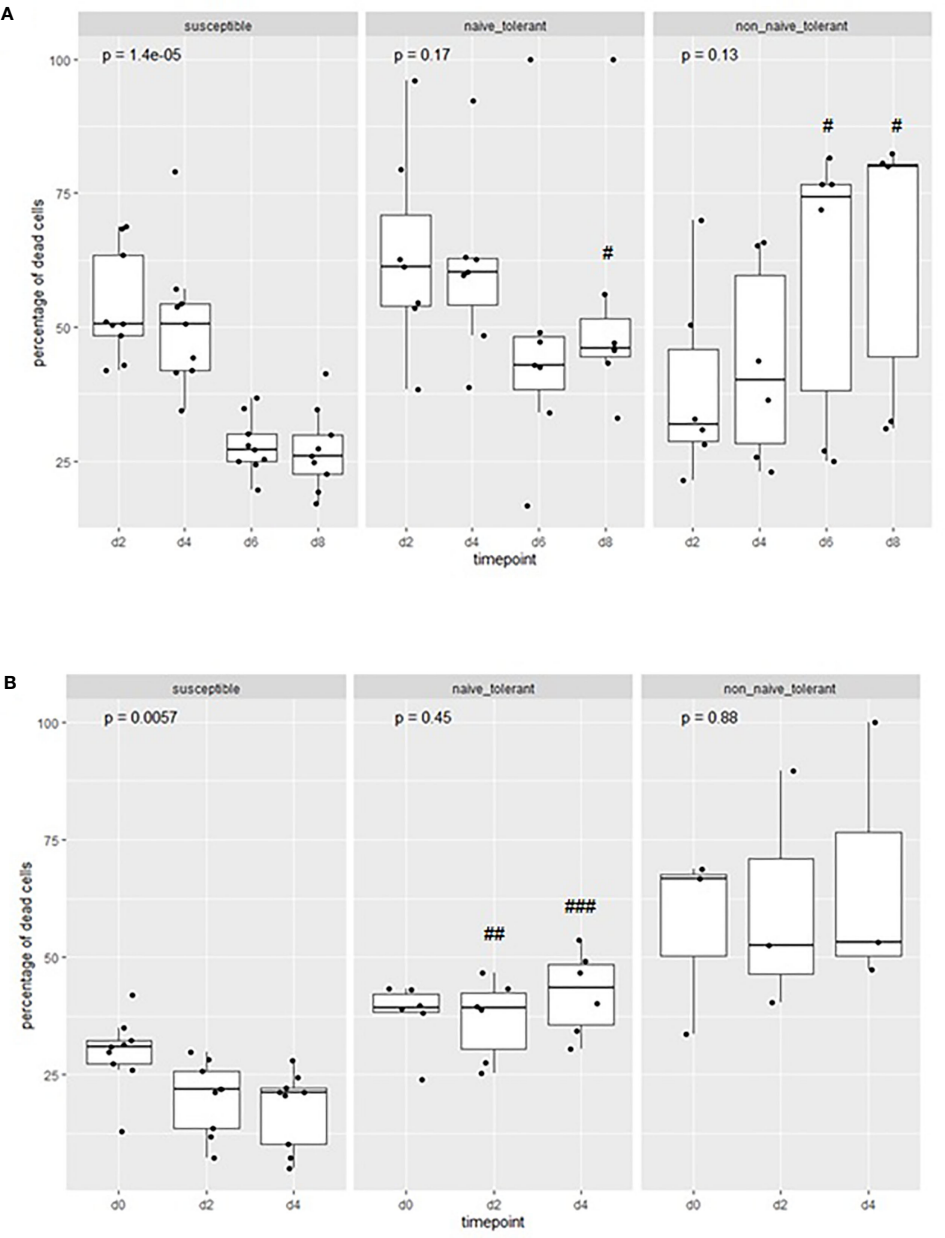
Proportions of dead cells in cultures from tolerant and susceptible animals: The percentage of dead cells in cultures of cells derived from tolerant and susceptible cattle during the first **(A)** and second **(B)** week of culture after infection *in vitro*. The p values represent the probability of a difference in the percentage of cells at different days within each group (Kruskal-Wallis test). The probability of a difference in percentage of dead cells at the same day between susceptible and tolerant populations is shown as ^#^p < 0.05; ^##^p <0.01; ^###^p< 0.001 (Wilcoxon test).

To quantitate the difference between surviving and susceptible animals, we estimated the percentage of dead cells during incubation of the cultures. The mean proportion of dead cells in cultures from susceptible animals decreased (**Figure 4**) during the incubation period, while that of the surviving animals remained relatively constant. The difference in the mean percentage of dead cells at day 8 was significantly different (p<0.05) between the naïve tolerant and susceptible cells. There were also significant differences between the two populations on day 2 (p<0.01) and day 4 p<0.001) of the second week. One cell line, from the surviving animal 4526, died out during the first week of infection (day 6).

Overall, the results indicate that there is an ongoing accumulation of dead cells in cultures from tolerant animals, which is greater than that in cultures from susceptible animals, and that the lower rate of expansion of cell numbers is not due solely to a lower rate of proliferation.

**Figure 5:**
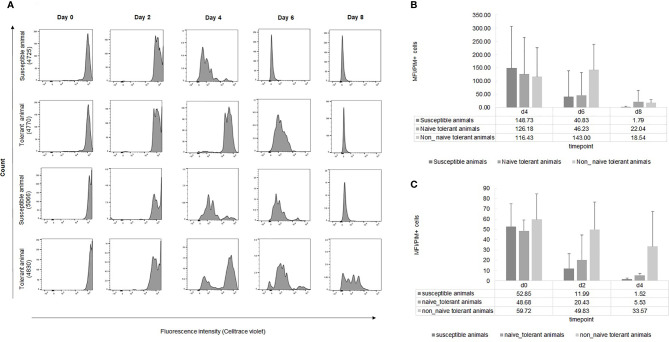
Dye dilution analysis of infected live cells from tolerant and susceptible animals. **(A)**: Representative examples of the division of live infected cells from seven tolerant and nine susceptible animals measured by CTV dye dilution during the first week of culture after infection *in vitro*. **(B**, **C)**: Results are presented as MFI of CTV of PIM+ cells at different time points during the first **(B)** and the second **(C)** week of infection. The bars represent STDEV.

An alternative method to measure the rate of cellular proliferation is by dye dilution analysis. We used CellTrace Violet (CTV) to compare the rates of proliferation in infected cell lines from surviving and susceptible cattle. Representative results from flow cytometry of cells from two surviving and two susceptible animals (**Figure 5A**) at different times during culture showed a more rapid loss of fluorescence in cells from the susceptible animals, indicating a higher rate of proliferation of these cells. This was confirmed by the more rapid decrease in the mean fluorescence intensity (MFI) of CTV per infected cell from the nine susceptible animals compared to the seven surviving ones (**Figures 5B, C**). The MFI of cells from susceptible animals had fallen to the detection limit by day 8, unlike that of cells from tolerant animals. A second aliquot of cells was incubated for a week before addition of CTV and as expected, the MFI of cells from surviving animals remained higher compared to that of susceptible cells.

#### Expression Levels of Inflammatory Cytokines Are Lower in WBC From Surviving Cattle

**Figure 6:**
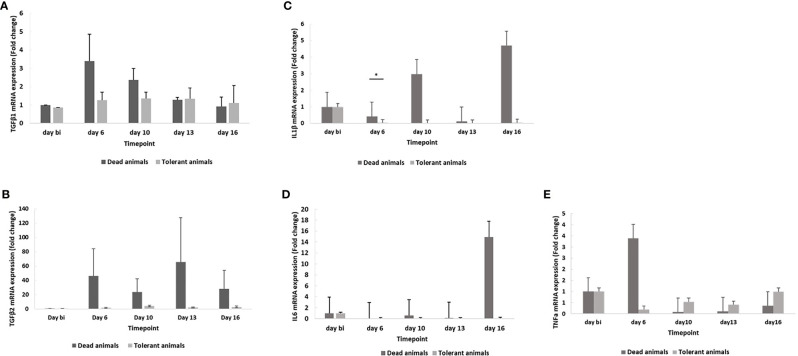
Expression of TGFβ1 **(A)**, TGFβ2 **(B)**, IL1β **(C)**, IL6 **(D)** and TNFα **(E)** mRNA in susceptible and tolerant cattle. The results were generated from total RNA isolated prior to infection (day bi: day before infection), day 6, day 10, day 13 and day 16 post-infection. The relative amounts of the cytokine transcripts were measured by real time qPCR. The GAPDH gene was used as internal control to normalize mRNA levels. The bars represent STDEV. *p < 0.05.

Because of their aberrant expression in *Theileria*-infected cells and the correlation with the severity of clinical signs in infected cattle (Yamada et al., 2009), we investigated the expression of the pro-inflammatory cytokines IL1β, IL6, TNFα, TGFβ1 and TGFβ2 in WBCs derived from seven surviving and seven susceptible animals. The WBCs were obtained prior to field exposure and on days 6, 10, 13 and 16 following exposure during the 2018 field study. The results show an increased level of IL1β expression on days 10 and 16 in susceptible cattle (**Figure 6C**), and of IL6 on day 16 (**Figure 6D**) and TNFα on day 6 (**Figure 6E**). Interestingly, these cytokines remain relatively unchanged in tolerant cattle. TGFβ1 expression also increases in susceptible animals, with peaks on days 6 and 10, whereas the expression in tolerant cattle remains essentially the same (**Figure 6A**). There was also a consistent increase in the expression of TGFβ2 in susceptible animals, with no observable change in tolerant animals (**Figure 6B**).

#### Proliferation of Cells From Surviving Cattle After Field Exposure

Cultures were established by infection *in vitro* of PBMCs isolated from surviving cattle after the last (2108) field study (**Supplementary Figures 1** and **2**) and examined for cellular proliferation. We noted very little increase in the numbers of live infected cells, compared to cells from surviving cattle taken before field exposure and cells from susceptible cattle (**Figures 2** and **3**). The difference was more marked in the second week of culture. Dye dilution analysis also indicated a lower rate of proliferation in the cells taken from the surviving cattle at the end of the field trial (**Figures 5B, C**). We attribute the difference between the cultures derived from cells taken before and after field infection to the generation of an acquired immune response in these animals and the activation of parasite-specific cytotoxic cells in culture.

We also managed to establish cell cultures *ex vivo* from one tolerant and one susceptible animal on day 16 following field exposure. The results (**Supplementary Figure 5**) reflected those from the cultures established by *in vitro* infection, in that the proliferation of cells from the tolerant animal was much less than that from the susceptible animal.

## Discussion

The progeny of sire 3167 show an inherited tolerance to natural infection with *T. parva*. Based on our *in vivo* and *in vitro* analyses, we propose that a decreased rate of expansion of infected lymphocytes, which are believed to be responsible for the pathology of the disease, is an important factor mediating the tolerance trait.

We believe this is the first report where tolerance to *T. parva* infection has been clearly demonstrated in a cohort descended from a single animal. Our identification of the 3167 sire line facilitated the field studies, by ensuring sufficient numbers of surviving animals within the number of animals that could be logistically included in the studies. The location of the study site was in an area known to offer heavy parasite challenge, as indicated by early acquisition of tick infestation and the uniformly rapid development of infection following introduction into the site. Nevertheless, progeny of bull 3167 most often survived, unlike unrelated animals.

We observed a significant delay in the detection of parasitosis in the surviving cattle and a decreased severity of the parasitosis in the three studies where this parameter was recorded. We consider that the corresponding delay in the onset and severity of pyrexia was due to the lower parasite load in the surviving cattle. This conclusion is supported by a detailed study of the kinetics of replication of *T. parva* that showed that the appearance of pyrexia was dependent on the number of macroschizont-infected cells, which the authors estimated to be 7 X 10^9^ infected cells per animal (Jarrett et al., 1969).

In line with the field observations, we have clear evidence that infected cells from surviving animals show a much reduced ability to proliferate *in vitro*, with the difference observable as early as six days after initiation of the cultures. We have recently generated evidence which indicates that there is no substantive difference in the ability of *T. parva* sporozoites to attach to and enter bovine lymphocytes (Chepkwony et al., in preparation). The proliferation results were supported by CTV dye dilution experiments, which also revealed a lower rate of proliferation in cells from surviving animals. Moreover, we observed a decreased expansion in a cell line established *ex vivo* from a tolerant animal compared to a susceptible animal. Although it is a very small sample size and the result could be confounded by the acquisition of acquired immunity in the tolerant animal, the *ex vivo* result is not at variance with the results from cell cultures established by infection *in vitro*.

Two lines of evidence suggest that the cells from surviving cattle undergo a phase of expansion which is subsequently arrested. First, infected cells are observed in the prescapular lymph nodes and subsequently in the parotid lymph nodes of tolerant cattle, albeit significantly later than in susceptible cattle. Second, live infected cells can be observed for at least two weeks in cultures established *in vitro* from cells from tolerant cattle, in the presence of an accumulating number of dead cells which presumably replace those which disintegrate. This latter observation suggests that there is continuous proliferation and death of the cells and that the difference in cell numbers is not due solely to a slower rate of cellular proliferation without an effect on cell viability.

The major pathology associated with *T. parva* infection is a severe pleuropneumonia resulting from the proliferation of lymphocytes infected with the macroschizont stage of the parasite (Irvin and Mwamachi, 1983). The association between the number of schizont-infected cells and the severity of the clinical reaction in infected animals has been observed previously under experimental conditions that allow variation in the infective dose of parasite administered to cattle (Jarrett et al., 1969). Thus, the time to onset of parasitosis and pyrexia in cattle decreased as the number of infected ticks applied to cattle increased. Similar results were obtained in experiments using parasite stabilates for infection, in which a longer time to death in animals receiving lower doses of infective material was also observed (Cunningham et al., 1974; Radley et al., 1974; Büscher et al., 1984). As in our studies, there was also a longer interval between the initial detection of parasites and of pyrexia to death in these animals.

A key question which arises from our studies is the molecular mechanism which mediates the decreased parasitosis in tolerant animals. We consider there are at least two possibilities. The first is an aberrant arrest in the proliferation mechanism of infected cells at some stage after initial infection and transformation, and the second involves the activation of an innate cytotoxic/suppressive cell population, *in vivo* and *in vitro*, in tolerant cattle. As outlined below, these possibilities are supported by previous studies on the biology of *T. parva*.

*Theileria-*induced transformation is strictly dependent on the presence of live parasites, and is fully reversible by treatment with parasiticidal compounds such as buparvaquone; drug-treated transformed leukocytes return to a quiescent, non-activated state (Dobbelaere et al., 2000; Hostettler et al., 2014). It has been shown that *Theileria* manipulates the host cell signaling pathways (Dobbelaere et al., 2000; Shiels et al., 2006) by upregulating genes involved in proliferation, such as the kinases PI3K/Akt (Baumgartner et al., 2000; Heussler et al., 2001; Haidar et al., 2015), JNK1/2 (Lizundia et al., 2007) and CK2 (Ole-MoiYoi et al., 1993; Dessauge et al., 2005). This results in constitutive activation of transcription factors including AP1 and NFκB (Guergnon et al., 2003; Schmuckli-Maurer et al., 2010). In parallel, there is increased expression of anti-apoptotic proteins such as cellular (c)-FLIP, which functions as a catalytically inactive form of caspase-8, and an X chromosome-linked inhibitor of apoptosis protein (IAP) as well as c-IAP, (Kuenzi et al., 2003). The increased expression of these proteins is rapidly reversed upon parasite elimination, and caspase-dependent apoptosis is generated by the caspase 9 and caspase 3 activation (Guergnon et al., 2003). To manipulate the signaling pathways, *Theileria* also hijacks host cell proteins (Haller et al., 2010; Schmuckli-Maurer et al., 2010; Latre de Late et al., 2019; Tretina et al., 2020).

The second option that could explain the reduced proliferation in tolerant animals is the involvement of an innate immune response. A recent study provided evidence for a role for NK cells in killing *T. parva*-infected lymphocytes (Connelley et al., 2014). In these experiments, conventional NKp46^+^CD3^-^cells and a novel, non-conventional NKp46^+^CD3^+^ T cell subset exhibited cytotoxicity towards *Theileria*-infected cell lines *in vitro*. The authors also observed an expansion of the NKp46^+^CD3^+^ population in some animals undergoing *T. parva* challenge, supporting an *in vivo* role for these cells in protection against infection.

Our results revealed an increase in the expression of genes encoding several inflammatory cytokine genes in susceptible cattle but not in those that survived. Previous studies in theileriosis have shown that inflammatory cytokines are closely associated with disease progression. Yamada et al. (2009) observed increased expression of IL1β, IL6 and TNFα in leukocytes from infected cattle, which coincided with the increase in the amount parasite DNA in peripheral blood. Preston et al. (1992) examined the effects of cytokines on *in vitro* growth of macroschizont-infected lymphocytes and noted increased proliferation in the presence of recombinant bovine TNFα. More recently, Guergnon et al. (2003) concluded that a TNFα autocrine loop increases the proliferation of and NF-κB transcriptional activity in *T. parva*-infected B cells (Guergnon et al., 2003). This effect was confirmed by using pharmacological or antibody-mediated inhibition of TNFα, both of which led to proliferation arrest. Moreover, earlier studies on resistance to *T. annulata* showed that infected macrophages from resistant *Bos indicus* (Sahiwal) cattle display lower levels of expression of genes associated with the inflammatory response compared to macrophages of susceptible *Bos taurus* (Holstein) cattle (Macguire et al., 2004; Glass and Jensen, 2007; Jensen et al., 2008; Glass et al., 2012). Confirmation of a direct causal role of any of these factors in the tolerance we have observed awaits further experimentation.

The heritable ability of cattle of the 3167 pedigree to survive a heavy field challenge with *T. parva* suggests that selection for animals carrying the responsible genes will reduce the burden imposed by Corridor disease and ECF. To that end, we have compared the genomes of the phenotyped progeny and unrelated animals to identify candidate regions responsible for the observed tolerance and which can be used as breeding markers (Wragg et al., submitted for publication).

In summary, the current studies demonstrate a clear, inherited capacity for some cattle to survive a heavy parasite challenge. There were highly significant differences between tolerant and susceptible cattle in the onset and severity of clinical signs and in the proliferative capacity of their infected cells *in vitro*. This difference, most likely due to the inherent level of multiplication of the parasitized cells or to the innate responses they induce, ultimately allows the resistant animals to survive and presumably generate a successful adaptive immune response. Further studies will be required to investigate these possibilities.

## Data Availability Statement

The original contributions presented in the study are included in the article/**Supplementary Material**. Further inquiries can be directed to the corresponding authors.

## Ethics Statement

The animal study was reviewed and approved by ILRI’s Institutional Animal Care and Use Committee.

## Author Contributions

Conceived, designed and conducted field studies: EC, GN, TS, PL, MC, SM, GP, RA, WM, and PT. Undertook genomic analyses to establish relatedness: DW and JP. Conceived and designed the *in vitro* experiments: PLdL and PT. Performed the *in vitro* experiments: PL. Analyzed the data: PLdL, GN, EC, and PT. Statistical analyses: EJP, DW, NN, and JP. Writing: Initial draft: PT, PLdL, DW, and JP. The cytokine study was performed by PLdL and AM. Review and editing: all authors. All authors contributed to the article and approved the submitted version.

## Funding

This research was funded in part by the Bill & Melinda Gates Foundation and with UK aid from the UK Foreign, Commonwealth and Development Office (Grant Agreement OPP1127286) under the auspices of the Centre for Tropical Livestock Genetics and Health (CTLGH), established jointly by the University of Edinburgh, SRUC (Scotland’s Rural College), and the International Livestock Research Institute. The findings and conclusions contained within are those of the authors and do not necessarily reflect positions or policies of the Bill & Melinda Gates Foundation nor the UK Government. Under the grant conditions of the Foundation, a Creative Commons Attribution 4.0 Generic License has already been assigned to the Author Accepted Manuscript version that might arise from this submission. This research was conducted as part of the CGIAR Research Program on Livestock. and we wish to acknowledge funding from the donors and organizations contributing to the CGIAR System that supported ILRI participation in this research (https://www.cgiar.org/funders). Some of the work described in this paper was supported by grant BB/H009515/1 awarded jointly by the then UK Department for International Development and the UK Biotechnology and Biological Sciences Research Council (BBSRC) under the Combating Infectious Diseases of Livestock for International Development (CIDLID) program.

## Conflict of Interest

The authors declare that the research was conducted in the absence of any commercial or financial relationships that could be construed as a potential conflict of interest.

## Publisher’s Note

All claims expressed in this article are solely those of the authors and do not necessarily represent those of their affiliated organizations, or those of the publisher, the editors and the reviewers. Any product that may be evaluated in this article, or claim that may be made by its manufacturer, is not guaranteed or endorsed by the publisher.
