# Supplementary material for: Inherited Tolerance in Cattle to the Apicomplexan Protozoan Theileria parva is Associated with Decreased Proliferation of Parasite-Infected Lymphocytes
Source: Front Cell Infect Microbiol. 2021 Nov 5;11:751671. doi: 10.3389/fcimb.2021.751671 (PMC8602341; doi:10.3389/fcimb.2021.751671)
Supplement: Supplementary file 1 [file DataSheet_1.docx]

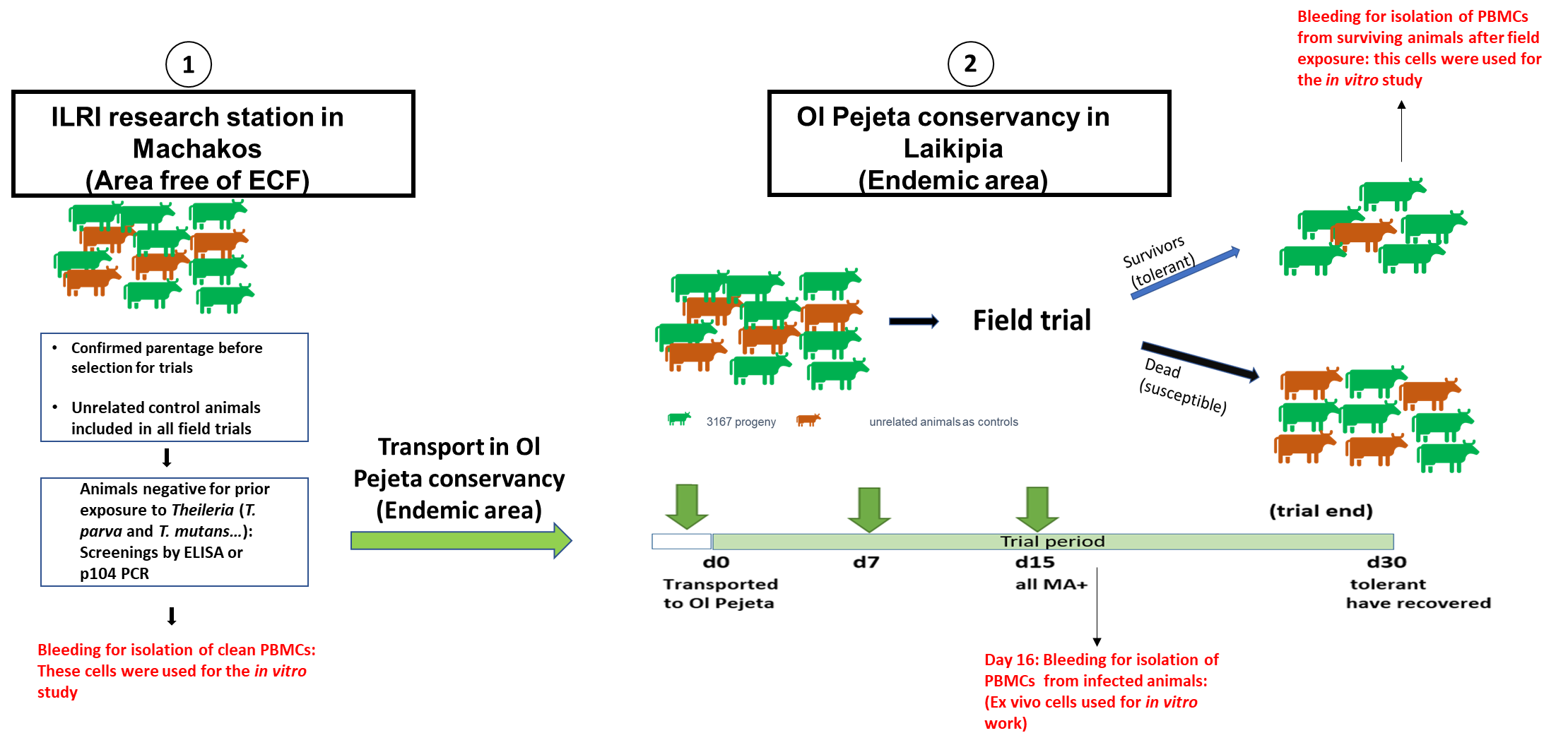


Supplementary Figure 1. Flow diagram for the field studies. (1) Animals were selected from the ILRI research station in Machakos, a region largely free of *T. parva*. The parentage was confirmed by genotyping, and unrelated animals were included as controls. Animals which were positive by *T. parva* by ELISA or p104PCR assays were excluded. (2) The animals were transported to the Ol Pejeta Conservancy (Kenya), which is considered endemic for *T. parva*. Parasitological and clinical data were collected during the field studies, as described. For the last field study (2018), PBMCs were collected before, during and after the field studies for the cytokine study and the *in vitro* proliferation assays.

**
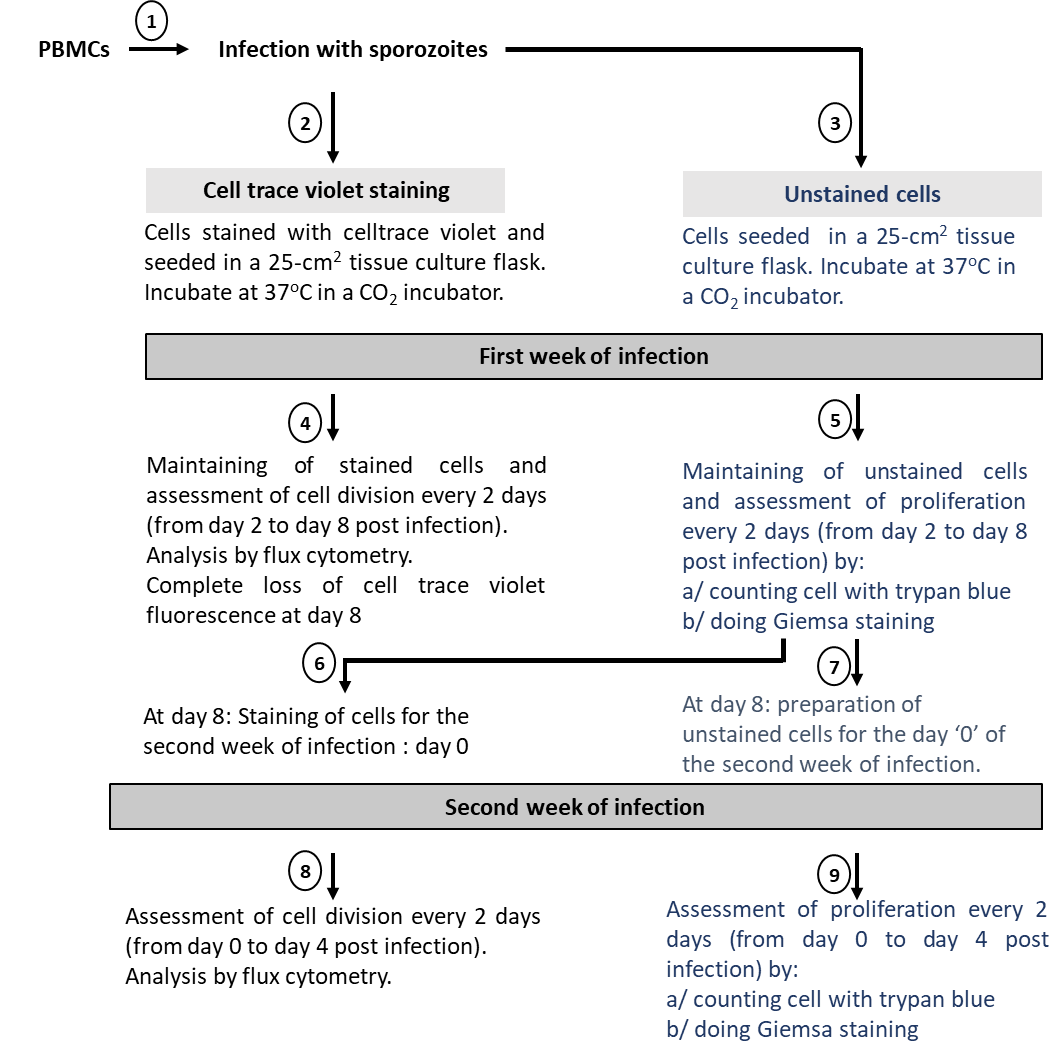
**

Supplementary Figure 2: Flow diagram for the proliferation assays: PBMCs were infected with sporozoites (1). Once infected, one lot of cells was seeded in T25 flask and maintained in culture (3), and another lot of cells was stained with CellTrace Violet (CTV) dye (2). During the first week of infection, assessment of proliferation was performed every 2 days for both stained cells (4) and unstained cells (5), allowing the results obtained with different techniques to be compared. Due to division of cells, the complete loss of CTV fluorescence was observed at day 8. To assess the proliferation during the second week of infection, some unstained cells maintained in culture were stained with CTV dye at day 8. This corresponds to day ‘0’ of the second week of infection. In parallel the same number of unstained cells was also prepared to compare the results between the different techniques.

Supplementary Table 1: Details of animals used to establish *in vitro* cell lines**.** The animals were part of the fifth field study in 2018. Sires 853, 1148 and 1456 are first generation offspring of bull 3167.

| Animals | Sire | Survival outcome |
| --- | --- | --- |
| 4851 | 1456 | Survived |
| 4830 | 1456 | Survived |
| 4687 | 1148 | Survived |
| 4807 | Unrel. | Survived |
| 5029 | Unrel. | Survived |
| 4526 | 1148 | Survived |
| 4770 | 1456 | Survived |
| 4725 | 1148 | Died, day 18 |
| 4795 | Unrel | Died, day 19 |
| 4819 | 1456 | Died, day 21 |
| 5066 | 853 | Died, day 17 |
| 4791 | 1456 | Died, day 20 |
| 4865 | 1456 | Died, day 22 |
| 4631 | 1148 | Euthanised, day 17 |
| 4858 | 1456 | Died, day 21 |
| 4774 | Unrel. | Died, day 20 |


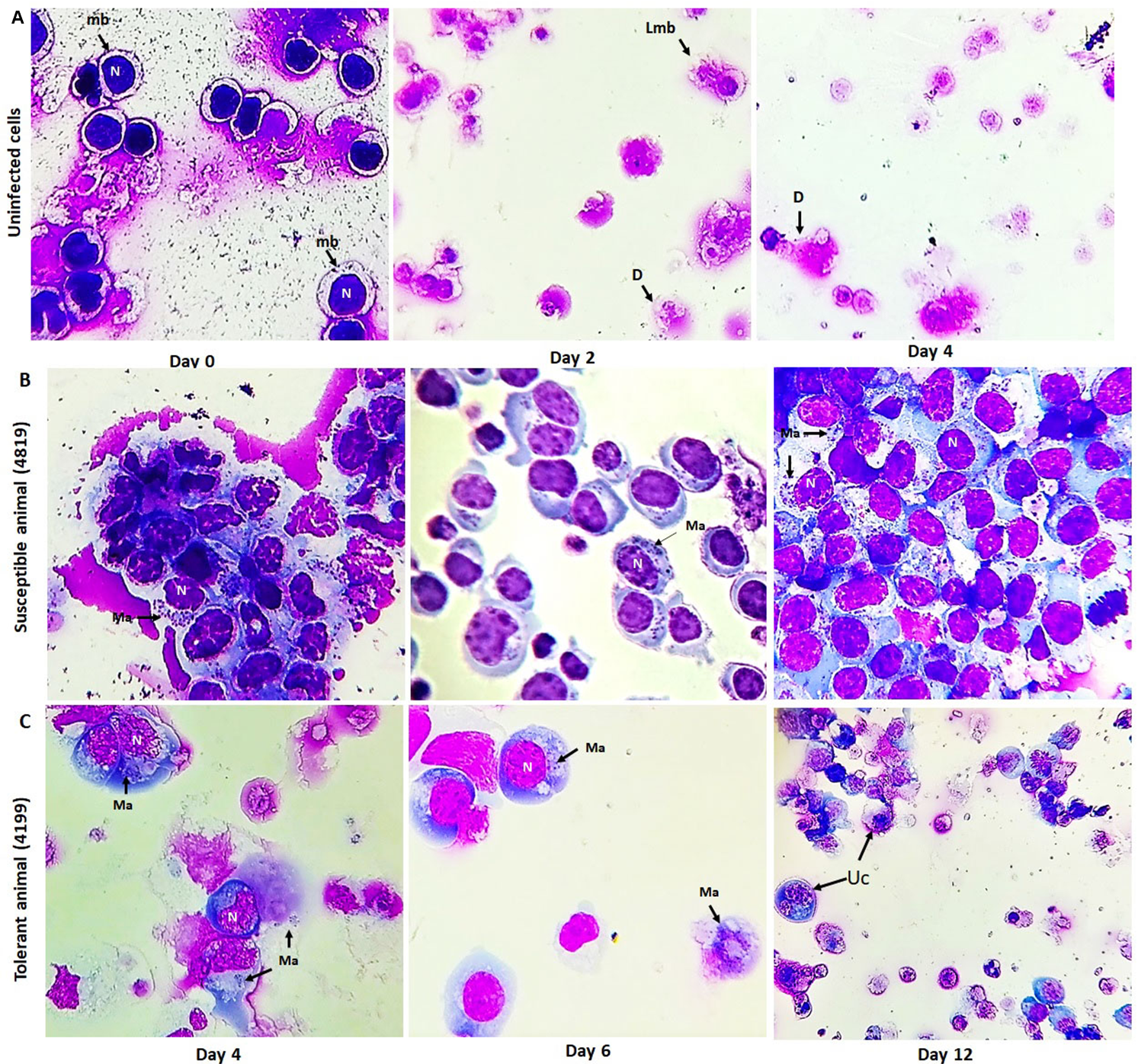


Supplementary Figure 3.**:** Giemsa-stained cells from cultures of uninfected and infected bovine lymphocytes at different time points during culture - Cells were removed from culture and 1.5 X 105 cells were deposited onto glass slides by Cytospin. The cells were fixed with methanol and stained with 10% of Giemsa solution for 30 min, air-dried and examined at magnification 50X. (**A):** Uninfected cells maintained in culture for 4 days, showing death and disintegration. (**B):** Infected cells from susceptible animal 4819 showing healthy multinucleated macroschizonts at day4, day 6 and day 12. (**C):** Infected cells from surviving animal 4199 showing fewer infected cells and evidence of host cell death. This is representative of different group of cells. **Hc:** Healthy cells; **Ma:** macroschizonts; **N:** nucleus of host cell; **mb:** membrane, **D**: debris; **Uc:** Unhealthy cells; **Lmb:** Loss of membrane integrity.


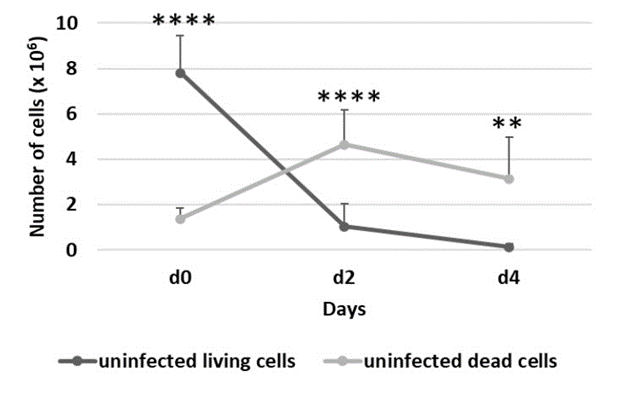


Supplementary Figure 4: Number of uninfected Live/ dead cells. Uninfected PBMCs were maintained in culture for 4 days. Staining with trypan blue allowed to determine the number of live/dead cells.


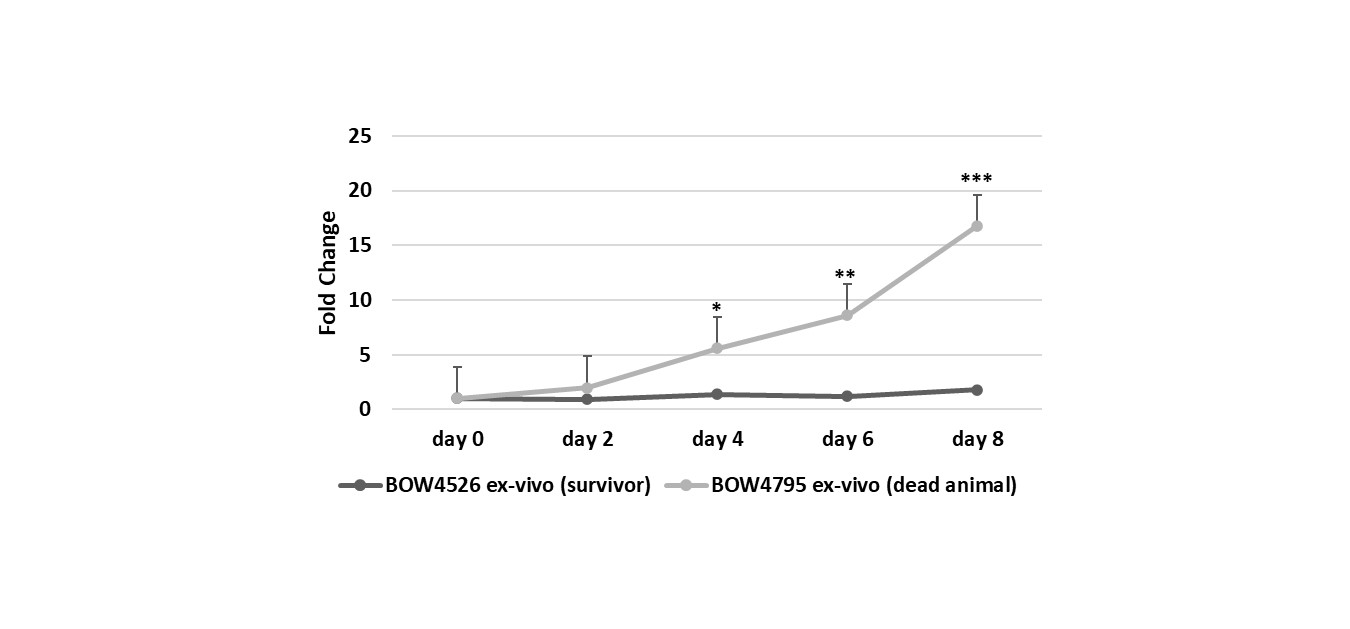


Supplementary Figure 5: Proliferation of ex vivo cells from infected susceptible and tolerant animals. Ex vivo cells obtained from infected tolerant (4526) or susceptible (4795) animals were maintained in culture for proliferation studies. The number of live cells were investigated every two days for 8 days. The results are the average of three experiments. Bars represent STDEV. The difference in number of live cells at each time point between susceptible and tolerant populations was calculated by a Student’s paired t-test, with a two-tailed distribution *: p < 0.05; **: p <0.01; ***: p< 0.001; ****: p<0.0001.
